# Broad-Spectrum Antibiotic Treatment and Subsequent Childhood Type 1 Diabetes: A Nationwide Danish Cohort Study

**DOI:** 10.1371/journal.pone.0161654

**Published:** 2016-08-25

**Authors:** Tine D. Clausen, Thomas Bergholt, Olivier Bouaziz, Magnus Arpi, Frank Eriksson, Steen Rasmussen, Niels Keiding, Ellen C. Løkkegaard

**Affiliations:** 1 Department of Gynecology and Obstetrics, Nordsjællands Hospital, University of Copenhagen, Hillerød, Denmark; 2 Department of Obstetrics, Rigshospitalet, University of Copenhagen, Copenhagen OE, Denmark; 3 Department of Public Health, Section of Biostatistics, University of Copenhagen, Copenhagen K, Denmark; 4 Laboratory MAP5, University Paris Descartes and CNRS, Sorbonne Paris Cité, Paris, France; 5 Department of Clinical Microbiology, Herlev Hospital, University of Copenhagen, Herlev, Denmark; 6 Department of Clinical Microbiology, Hvidovre Hospital, University of Copenhagen, Hvidovre, Denmark; King Abdullah International Medical Research Center, SAUDI ARABIA

## Abstract

**Background:**

Studies link antibiotic treatment and delivery by cesarean section with increased risk of chronic diseases through changes of the gut-microbiota. We aimed to evaluate the association of broad-spectrum antibiotic treatment during the first two years of life with subsequent onset of childhood type 1 diabetes and the potential effect-modification by mode of delivery.

**Materials and Methods:**

A Danish nationwide cohort study including all singletons born during 1997–2010. End of follow-up by December 2012. Four national registers provided information on antibiotic redemptions, outcome and confounders. Redemptions of antibiotic prescriptions during the first two years of life was classified into narrow-spectrum or broad-spectrum antibiotics. Children were followed from age two to fourteen, both inclusive. The risk of type 1 diabetes with onset before the age of 15 years was assessed by Cox regression. A total of 858,201 singletons contributed 5,906,069 person-years, during which 1,503 children developed type 1 diabetes.

**Results:**

Redemption of broad-spectrum antibiotics during the first two years of life was associated with an increased rate of type 1 diabetes during the following 13 years of life (HR 1.13; 95% CI 1.02 to 1.25), however, the rate was modified by mode of delivery. Broad-spectrum antibiotics were associated with an increased rate of type 1 diabetes in children delivered by either intrapartum cesarean section (HR 1.70; 95% CI 1.15 to 2.51) or prelabor cesarean section (HR 1.63; 95% CI 1.11 to 2.39), but not in vaginally delivered children. Number needed to harm was 433 and 562, respectively. The association with broad-spectrum antibiotics was not modified by parity, genetic predisposition or maternal redemption of antibiotics during pregnancy or lactation.

**Conclusions:**

Redemption of broad-spectrum antibiotics during infancy is associated with an increased risk of childhood type 1 diabetes in children delivered by cesarean section.

## Introduction

Infectious morbidity and mortality have been reduced dramatically since the introduction of penicillin and other antibiotics. However, expanding use of antibiotics has unwanted ecological side-effects. Recent studies indicate that antibiotic treatment may influence the human organism in a long-term perspective and increase the risk of chronic diseases.[1–3] It is suggested that the disease-causing effect of antibiotics works through changes of the gut-microbiota, and broad-spectrum antibiotics are thought to have the most prominent effect.[2]

Since 1997, the incidence rate of type 1 diabetes in 0–4 year old children in Denmark has stabilized at 14 new cases per 100,000 person-years, whereas the incidence rates among 5–9 and 10–14 year old children have been steadily increasing.[4] Studies have linked microbiomic changes to an increased risk of type 1 diabetes, through a complex disturbance of the maturation of the immune system and an increased vulnerability to environmental triggers of autoimmunity.[2;5] As the microbiota is affected for several months following a broad-spectrum antibiotic treatment,[6] it has been intriguing to link the increasing incidence of childhood type 1 diabetes to the rising use of broad-spectrum antibiotics. Published studies show inconsistent results.[5;7–11] Mode of delivery has also been linked to an increased risk of type 1 diabetes, possibly due to microbiomic changes, but findings from studies are conflicting. [4;12–15] According to the “hygiene hypothesis” exposure to microbes, including those in the genital tract during birth, increases the microbial biodiversity and is thought to be beneficial for the immune system maturation and protective for later development of a variety of diseases. Therefore, harmful effects of broad-spectrum antibiotics would be expected to be most pronounced among children delivered by prelabor cesarean section, whom had not been exposed to the maternal vaginal flora.[1;2]

We evaluated the association of broad-spectrum antibiotic treatment during the first two years of life with subsequent onset of childhood type 1 diabetes and explored potential effect-modification by mode of delivery.

## Materials and Methods

We established a database based on four Danish nationwide registers: the Medical Birth Registry (1997 to 2010),[16] the Fertility Database (1997 to 2010),[17] the National Patient Registry (1977 to 2011),[18] the Register of Medicinal Product Statistics (1997 to 2012)[19] and additional information from Statistics Denmark (1997 to 2012).

By a unique personal identification number, linkage between registers and between children and their parents was possible.[20] The Danish Data Protection Agency approved the study (Journal number: 2012-58-0004). After linkage data were de-identified to ensure data-safety.

The Medical Birth Registry provided data on date of birth, vital status at birth, mode of delivery, parity, multiple births, birth weight and gestational age at birth.

The Fertility Database linked children to their parents and provided data on offspring sex, and date of birth for the parents.

The National Patient Registry, delivered hospital admission dates, discharge diagnoses for diabetes in the child, mother and father, and codes for diagnoses and surgical procedures related to mode of delivery.

The Register of Medicinal Product Statistics, valid on the individual level since 1997, captured redeemed prescriptions on insulin/insulin analogues and oral antidiabetics for the child, mother and father and on antibiotics for the child and the mother.

The relevant codes and changes over time for diagnoses and procedures are listed in S1 Table.

Statistics Denmark provided information on vital status, emigration and parental educational status.

### Study population

All live-born children in Denmark from 1 January 1997 through 31 December 2010 (n = 912,797) were identified in the Medical Birth Registry. We excluded 38,218 children from multiple pregnancies (n = 37,895) and pregnancies with errors in the personal identification number (n = 323). Furthermore, we excluded 16,378 children with events before their two years birthday due to either death (n = 3,412), emigration (n = 12,790) or diagnosis of type 1 diabetes (n = 176). The final population included 858,201 live-born singleton children born to 527,927 mothers.

**Fig 1:**
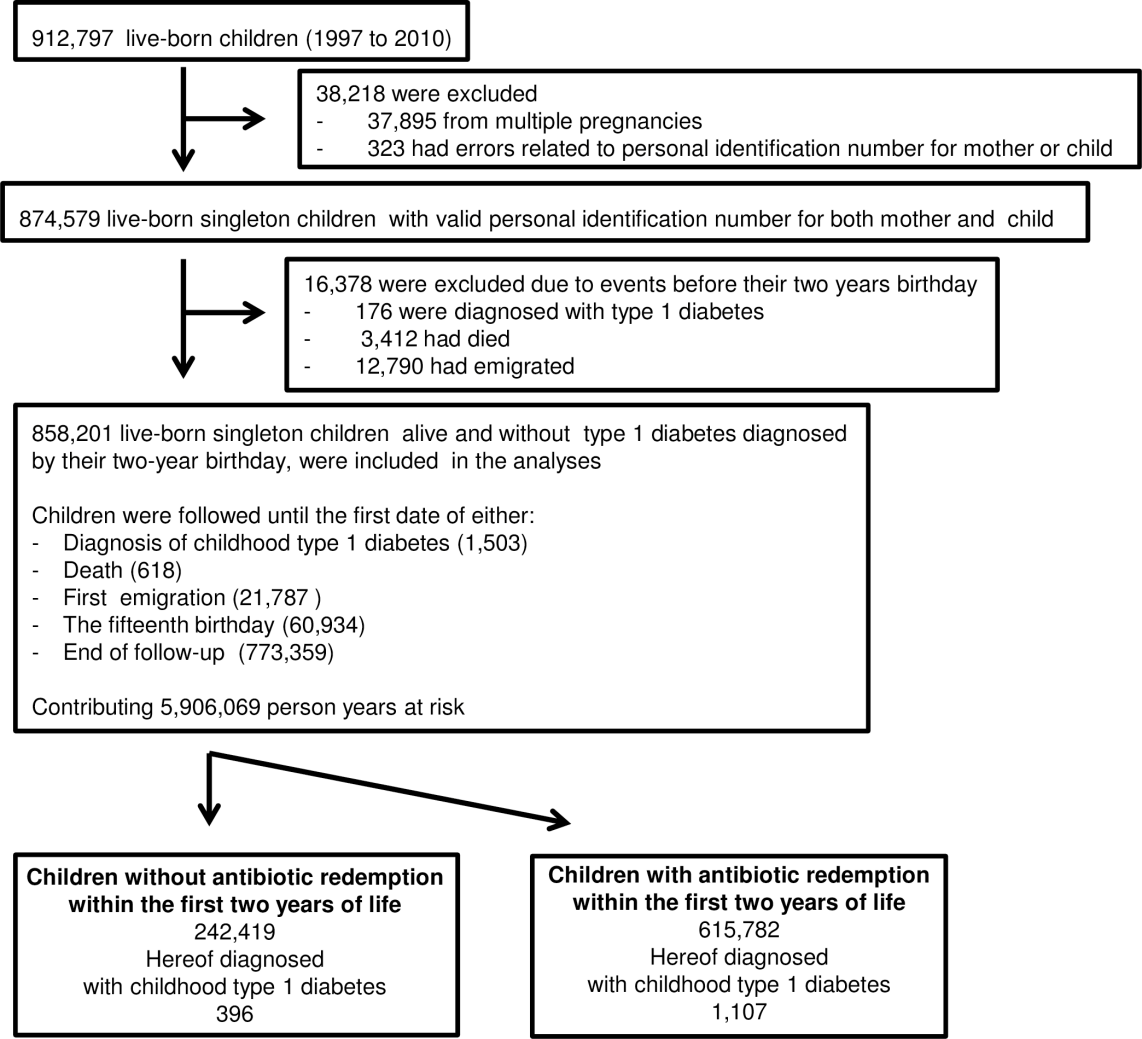
Inclusion, exclusion and data censoring. Shown are the number of children who met the exclusion criteria, the number of events and the distribution of antibiotics redemption and diagnosis of childhood type 1 diabetes.

The children were followed from age two until their fifteenth birthday (n = 60,934), the date of type 1 diabetes diagnosis (n = 1,503), death (n = 618), first emigration (n = 21,787) or end of follow-up by December 2012 (n = 773,359), whichever came first (Fig 1).

### Exposure

Outpatient redemptions of antibiotic prescriptions for the child during the first two years of life were classified into either: any type of antibiotics (yes or no), narrow-spectrum antibiotics (yes or no) or broad-spectrum antibiotics (yes or no), classified in accordance with the Danish Integrated Antimicrobial Monitoring and Research Program 2013 (S2 Table).[21] Consequently, children exposed to both narrow-and broad spectrum antibiotics, would be classified as exposed in all analyses evaluating effects of any type, narrow-spectrum or broad-spectrum antibiotics, respectively, whereas children treated with only broad-spectrum antibiotics, would be included in the exposed group in analyses evaluating effects of any type and broad-spectrum antibiotics but in the un-exposed group, when evaluating the effect of narrow-spectrum antibiotics. The classification into the three different exposure groups were used in separate analyses.

### Outcome variable

We followed children during 13 years from age two to fourteen, both inclusive, contributing 5,906,069 person-years at risk and an average duration of follow-up of 6.9 years. We defined childhood type 1 diabetes in children who before the age of fifteen fulfilled one of two of the following criteria or both. Either they were discharged from a hospital with a diabetes diagnosis (type 1 or unspecified) or they had redeemed at least two prescriptions on insulin/insulin analogues within one year. Children who additionally redeemed two prescriptions on oral anti-diabetics within one year were considered having type 2 diabetes. The date of onset of type 1 diabetes was defined as the first of either the date of the first diabetes-related hospital admission or the date for the second redemption of insulin/insulin analogues (S1 Table).

### Confounders and other covariates

We based selection of possible confounders on knowledge from previous studies on risk factors for type 1 diabetes, as well as on theoretical considerations regarding factors known to be associated with both the use of antibiotics and risk of type 1 diabetes.[22] Confounders comprised birth year (1997–2010), sex (male or female), parity (primiparous or multiparous) and mode of delivery (vaginal delivery, intrapartum cesarean section or prelabor cesarean section) (S1 Table).

Included parental confounders were genetic predisposition measured as maternal or paternal diabetes status at childbirth, as well as maternal and paternal educational level and age (<25, 25–30 or >30 years) at childbirth. Maternal and paternal type 1 diabetes was defined in parents discharged since 1977 with a diabetes diagnosis (type 1 or unspecific). Parents with two prescriptions on oral antidiabetics within one year were interpreted as having type 2 diabetes. The onset date of type 1 diabetes was defined as the date for the first diabetes-related hospital admission. The variable was classified into two categories (type 1 diabetes diagnosed before childbirth: yes or no). Education was recorded as the highest level of ongoing or finished education and classified into three categories (elementary school/high school, short education/skilled worker or medium/long education). Indicators (yes or no) on maternal redemption of antibiotics during pregnancy (Trimester 1, 2 or 3) and during the first six months after delivery were derived for any, narrow-spectrum or broad-spectrum antibiotics.

Birth weight (<2,500, 2,500–4,000 or >4,000 grams) and gestational age (<34, 34–36, 37–40 or >40 weeks gestation) were available.

### Data analysis

We estimated childhood type 1 diabetes hazard ratios (HRs) for redemption of antibiotics using a Cox proportional hazards model, using the survival package (version 2.37–7) in R (version 3.0.2). The proportional hazards assumption of the Cox regression model implies that the association with antibiotics does not change with age. To examine the validity of this assumption, we fitted models where we allowed the association to change in three-year intervals. These models validated that the proportional hazards assumption was met.

Robust (sandwich) standard errors were used to adjust for multiple births from the same woman. To examine the impact of different confounders we conducted partially adjusted models as well as fully adjusted models including all confounders (year of birth, mode of delivery, maternal- and paternal type 1 diabetes status at childbirth, maternal- and paternal age at childbirth, maternal- and paternal educational level at childbirth, offspring sex, parity). Missing covariates were handled by complete case analysis.

Additional models evaluated the potential impact of gestational age and birth weight. We explored the potential interaction between antibiotic redemption and delivery mode, parental type 1 diabetes status at childbirth, parity and maternal redemption of antibiotics during pregnancy and during the first 6 months after delivery by adding an interaction term to the model. To illustrate the effect of mode of delivery on the number of children, who on average should be exposed to broad-spectrum antibiotics during the first two years of life to cause childhood type 1 diabetes in one child (diagnosed from two to fifteen years of age), we calculated the number needed to harm for different subgroups of mode of delivery (all deliveries, intrapartum cesarean section and prelabour cesarean section, respectively).[23]

Finally, potential effects of specific categories of antibiotics were evaluated in analyses of children exposed to the following antibiotic sub-categories based on ATC-codes: J01CA (Extended-spectrum penicillin), J01CR (Combined penicillins, including beta-lactamase inhibitors), J01CE (Beta-lactamase sensitive penicillins), and J01FA (Macrolides).

## Results

In the final population of 858,201 singleton children born in Denmark during 1997 to 2010 on average 615,782 children (71.8%) had redeemed antibiotics during the first two years of life. Hereof, 440,006 children (51.3%) had redeemed narrow-spectrum antibiotics and 441.273 (51.4%) broad-spectrum antibiotics, as 174,509 (20.3%) had redeemed only narrow-spectrum antibiotics, 175,776 (20,5%) only broad-spectrum antibiotics and 265.497 (30.9%) both narrow- and broad-spectrum antibiotics (Table 1).

**Table 1:** Background characteristics for singleton children born 1997 to 2010 and their parents^a^.

		Children who within the first two years of life redeemed			
		No	Any	Narrow-spectrum	Broad-spectrum
		Antibiotics	Antibiotics	Antibiotics	Antibiotics
Variable	All children, n	n (%^b^)	n (%^b^)	n (%^b^)	n (%^b^)
**Number of children**	858,201	242,419 (28.2)	615,782 (71.8)	440,006 (51.3)	441,273 (51.4)
**Mode of delivery**					
** Vaginal**	707,254	204,004 (28.8)	503,250 (71.2)	359,236 (50.8)	358,375 (50.7)
** Intrapartum cesarean section**	71,241	18,604 (26.1)	52,637 (73.9)	37,763 (53.0)	38,455 (54.0)
** Prelabor cesarean section**	79,706	19,811 (24.9)	59,895 (75.1)	43,007 (54.0)	44,443 (55.8)
**Sex**					
** Female**	417,906	131,245 (31.4)	286,661 (68.6)	200,122 (47.9)	202,809 (48.5)
** Male**	440,295	111,174 (25.2)	329,121 (74.8)	239,884 (54.5)	238,464 (54.2)
**Parity**					
** Multiparous**	480,714	133,927 (27.9)	346,787 (72.1)	2503,16 (52.1)	247,621 (51.5)
** Primiparous**	371,012	106,710 (28.8)	264,302 (71.2)	186,728 (50.3)	189,912 (51.2)
** Missing**	6,475	1,782 (27.5)	4,693 (72.5)	2,962 (45.7)	3,740 (57.8)
**Birth weight (grams)**					
** <2,500**	28,637	7,039 (24.6)	21,598 (75.4)	15,422 (53.9)	16,103 (56.2)
** 2,500–4,000**	670,334	190,169 (28.4)	480,165 (71.6)	342,049 (51.0)	344,305 (51.4)
** >4,000**	152,888	43,084 (28.2)	109,804 (71.8)	79,547 (52.0)	77,917 (51.0)
** Missing**	6,342	2,127 (33.5)	4,215 (66.5)	2,988 (47.1)	2,948 (46.5)
**Gestational age (weeks)**					
** <34**	10,616	2,386 (22.5)	8,230 (77.5)	5,945 (56.0)	6,193 (58.3)
** 34–36**	30,314	7,459 (24.6)	22,855 (75.4)	16,625 (54.8)	16,953 (55.9)
** 37–40**	586,421	163,891 (27.9)	422,530 (72.1)	3018,59 (51.5)	303,460 (51.7)
** >40**	226,583	67,144 (29.6)	159,439 (70.4)	113,642 (50.2)	112,763 (49.8)
** Missing**	4,267	1,539 (36.1)	2,728 (63.9)	1,935 (45.3)	1,904 (44.6)
**Paternal age**^**c**^ **(years)**					
** <25**	34,837	8,509 (24.4)	26,328 (75.6)	19,085 (54.8)	19,051 (54.7)
** 25–30**	227,259	60,589 (26.7)	166,670 (73.3)	119,979 (52.8)	120,657 (53.1)
** >30**	587,109	170,498 (29.0)	416,611 (71.0)	296,573 (50.5)	297,149 (50.6)
** Missing**	8,996	2,823 (31.4)	6,173 (68.6)	4,369 (48.6)	4,416 (49.1)
**Maternal age**^**c**^ **(years)**					
** <25**	83,259	20,919 (25.1)	62,340 (74.9)	45,187 (54.3)	45,238 (54.3)
** 25–30**	325,923	88,835 (27.3)	237,088 (72.7)	170,668 (52.4)	170,698 (52.4)
** >30**	449,019	132,665 (29.5)	316,354 (70.5)	224,151 (49.9)	225,337 (50.2)
**Paternal educational level**^**c**^					
** Elementary school/high school**	202,407	53,343 (26.4)	149,064 (73.6)	107,581 (53.2)	107,837 (53.3)
** Short education/skilled worker**	405,022	108,966 (26.9)	296,056 (73.1)	212,426 (52.4)	213,364 (52.7)
** Medium/long education**	221,690	71,235 (32.1)	150,455 (67.9)	105,528 (47.6)	105,605 (47.6)
** Missing**	29,082	8,875 (30.5)	20,207 (69.5)	14,471 (49.8)	14,467 (49.7)
**Maternal educational level**^**c**^					
** Elementary school/high school**	215,134	56,661 (26.3)	158,473 (73.7)	115,457 (53.7)	113,664 (52.8)
** Short education/skilled worker**	314,776	82,694 (26.3)	232,082 (73.7)	167,095 (53.1)	167,691 (53.3)
** Medium/long education**	310,730	97,136 (31.3)	213,594 (68.7)	149,443 (48.1)	151,664 (48.8)
** Missing**	17,561	5,928 (33.8)	11,633 (66.2)	8,011 (45.6)	8,254 (47.0)
**Paternal type 1 diabetes**^**d**^					
** No**	844,823	238,360 (28.2)	606,463 (71.8)	433,390 (51.3)	434,590 (51.4)
** Yes**	4,382	1,236 (28.2)	3,146 (71.8)	2,247 (51.3)	2,267 (51.7)
** Missing**	8,996	2,823 (31.4)	6,173 (68.6)	4,369 (48.6)	4,416 (49.1)
**Maternal type 1 diabetes**^**d**^					
** No**	855,33	241,699 (28.3)	613,631 (71.7)	438,451 (51.3)	439,683 (51.4)
** Yes**	2,871	720 (25.1)	2,151 (74.9)	1,555 (54.2)	1,590 (55.4)

^a^Reported in all children and classified regarding redemption of antibiotics within the first two years of life (n = 858,201).

Included were children who by their two years birthday were alive and had not emigrated or been diagnosed with type 1 diabetes

^b^Percentage of all children (row percentage)

^c^At childbirth

^d^Diagnosed before childbirth

**Fig 2:**
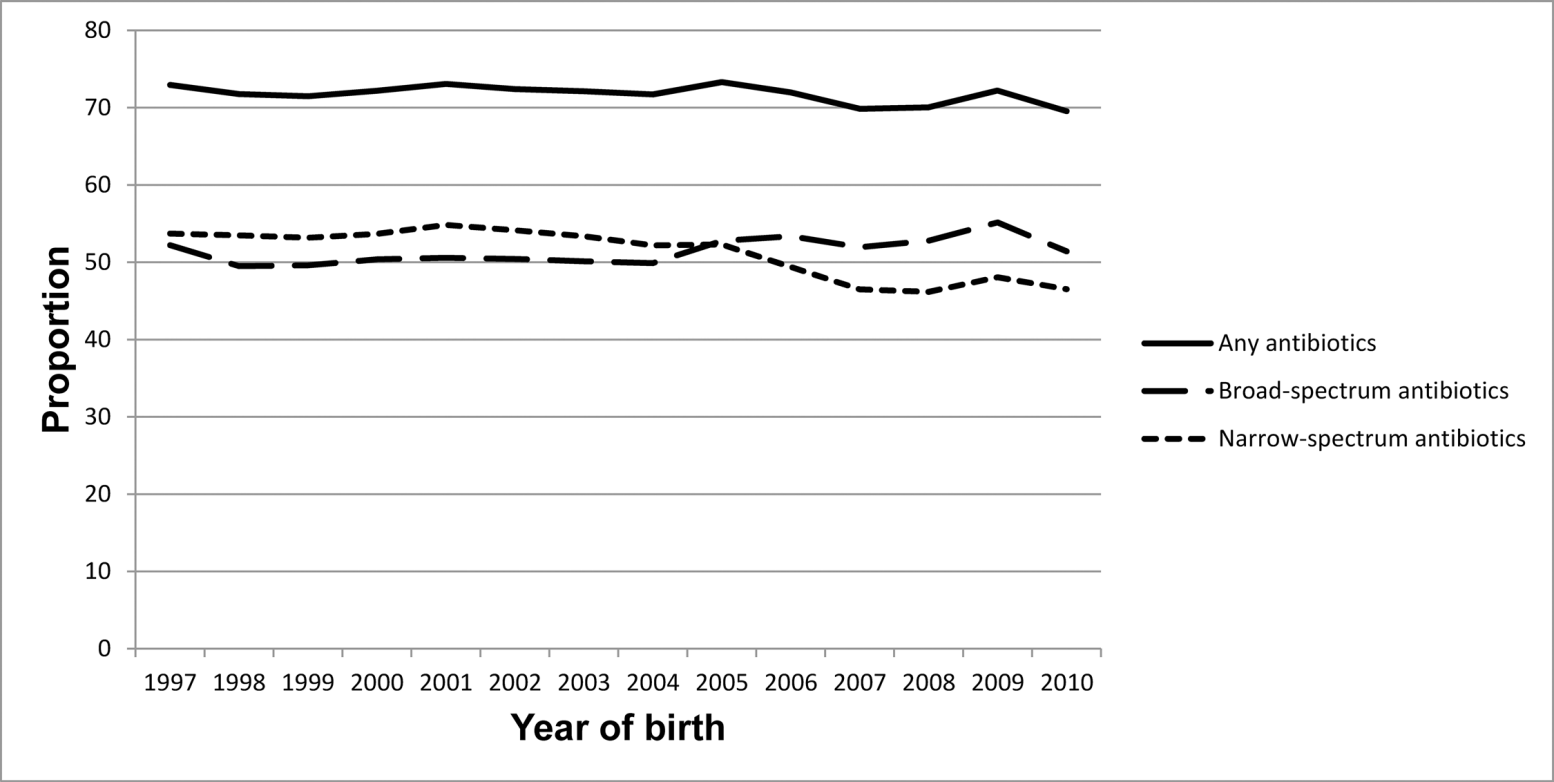
Redemption of antibiotics within the first two years of life. Included are all children born in Denmark 1997 to 2010, who by their two years birthday were alive and had not emigrated or been diagnosed with type 1 diabetes (n = 858,201). The proportion of children redeeming broad-spectrum and narrow-spectrum antibiotics, respectively, include also children who had redeemed both broad- and narrow-spectrum antibiotics within the first two years of life.

The overall rate of children who redeemed any type of antibiotics within the first two years of life was stable in the birth cohorts from 1997 to 2010. However, since 2005 broad-spectrum antibiotics were more frequently redeemed than narrow-spectrum antibiotics (Fig 2).

Redemption of antibiotics during the first two years of life was most prominent in children delivered by cesarean section, males, children with a birth weight below 2,500g or gestational age below 34 weeks, children having young parents with a short education, as well as children delivered by mothers with type 1 diabetes diagnosed before childbirth (Table 1).

Type 1 diabetes was diagnosed in 1,503 children (Fig 1).

In crude analyses, children who had redeemed prescriptions on any type of antibiotics during the first two years of life had a comparable rate of childhood type 1 diabetes, to children without redemptions of antibiotics (HR 1.07; 95% CI 0.96 to 1.20). Estimates remained essentially unchanged when adjusted for year of birth (HR 1.07; 95% CI 0.95 to 1.20) and mode of delivery (HR 1.07; 95% CI 0.95 to 1.20), as well as after further adjustment for parental age, education and type 1 diabetes status at childbirth, parity and sex (HR 1.06; 95% CI 0.94 to 1.19, Table 2). The same pattern was found regarding narrow-spectrum antibiotics. In contrast, children who had redeemed prescriptions on broad-spectrum antibiotics had a higher rate of childhood type 1 diabetes as compared to children who did not, in crude (HR 1.14; 95% CI 1.03 to 1.26) as well as in adjusted analyses (HR 1.13; 95% CI 1.02 to 1.25, Table 2). Apart from broad-spectrum antibiotics the following predictors of type 1 diabetes was found: Primiparity (HR 1.12; 95% CI 1.00 to 1.26), paternal type 1 diabetes diagnosed before childbirth (HR 11.21; 95% CI 8.85 to 14.20) and maternal type 1 diabetes diagnosed before childbirth (HR 6.19; 95% CI 4.31 to 8.91). The association with maternal education (Short education/skilled worker: HR 1.13; 95% CI 0.99 to 1.29) did not reach statistical significance. Further adjustment for birth weight or gestational age did not change estimates (S3 Table). The association between antibiotics during the first two years of life and type 1 diabetes was strongly modified by mode of delivery (P-value for the interaction term between broad-spectrum antibiotics and mode of delivery: 0.0023). In vaginally delivered children redemption of antibiotics, regardless of type, was not associated with an increased rate of childhood type 1 diabetes. In contrast, an association with broad-spectrum antibiotics was found among children delivered by intrapartum cesarean section (HR 1.70; 95% CI 1.15 to 2.51) as well as by prelabor cesarean section (HR 1.63; 95% CI 1.11 to 2.39). In children delivered by prelabor cesarean section redemption of antibiotics of any type was associated with a two-fold increased rate of childhood type 1 diabetes (HR 1.91; 95% CI 1.14 to 3.20). Regardless of mode of delivery, narrow-spectrum antibiotics were not associated with childhood type 1 diabetes (Table 3).

**Table 2:** Associations between redemption of antibiotics within the first two years of life and subsequent onset of childhood type 1 diabetes (2 to 14 years, both included).

	Children who within the first two years of life redeemed		
	Any	Narrow-spect.	Broad-spect.
	Antibiotics	Antibiotics	Antibiotics
	Fully adjusted model^a^	Fully adjusted model^a^	Fully adjusted model^a^
Variable	HR (95% CI)	HR (95% CI)	HR (95% CI)
**Number of children**	810,946	810,946	810,946
**Number of observed events**	1,442	1,442	1,442
**Redemption of antibiotics**			
**within two years of life**			
** No**	1.00	1.00	1.00
** Yes**	1.06 [0.94;1.19]	1.02 [0.92;1.13]	1.13 [1.02;1.25]
**Mode of delivery**			
** Vaginal**	1.00	1.00	1.00
** Intrapartum cesarean section**	0.97 [0.80;1.17]	0.97 [0.80;1.18]	0.96 [0.79;1.17]
** Prelabor cesarean section**	0.99 [0.82;1.20]	0.99 [0.82;1.20]	0.99 [0.81;1.19]
**Sex**			
** Female**	1.00	1.00	1.00
** Male**	0.94 [0.84;1.04]	0.94 [0.85;1.04]	0.93 [0.84;1.04]
**Parity**			
** Multiparous**	1.00	1.00	1.00
** Primiparous**	1.12 [1.00; 1.26]	1.12 [1.00;1.25]	1.12 [1.00;1.26]
**Paternal age (years)**^**b**^			
** 0–24**	1.05 [0.79;1.40]	1.05 [0.79;1.40]	1.05 [0.79;1.40]
** 25–30**	1.00	1.00	1.00
** 31–100**	0.95 [0.83;1.08]	0.95 [0.83;1.08]	0.95 [0.83;1.08]
**Maternal age (years)**^**b**^			
** 0–24**	0.99 [0.80;1.23]	0.99 [0.80;1.23]	0.99 [0.80;1.22]
** 25–30**	1.00	1.00	1.00
** 31–100**	1.04 [0.92;1.18]	1.04 [0.92;1.18]	1.04 [0.92;1.18]
**Paternal education**^**b**^			
** Elementary school/high school**	1.00	1.00	1.00
** Short education/skilled worker**	1.02 [0.90;1.16]	1.02 [0.90;1.16]	1.02 [0.90;1.16]
** Medium/long education**	0.95 [0.80;1.12]	0.94 [0.80;1.11]	0.95 [0.80;1.12]
**Maternal education**^**b**^			
** Elementary school/high school**	1.00	1.00	1.00
** Short education/skilled worker**	1.13 [0.99;1.29]	1.13 [0.99;1.29]	1.13 [0.99;1.29]
** Medium/long education**	1.02 [0.87;1.20]	1.02 [0.87;1.19]	1.02 [0.87;1.20]
**Paternal type 1 diabetes**^**c**^			
** No**	1.00	1.00	1.00
** Yes**	11.20 [8.84;14.19]	11.20 [8.84;14.19]	11.21 [8.85;14.20]
**Maternal type 1 diabetes**^**c**^			
** No**	1.00	1.00	1.00
** Yes**	6.20 [4.31;8.91]	6.20 [4.31;8.91]	6.19 [4.31;8.91]

Hazard ratio (HR) for type 1 diabetes diagnosed in children before 15 years of age are reported with 95% confidence interval (95% CI).

^a^Fully adjusted model adjusted for: Year of birth, mode of delivery, sex, parity, maternal and paternal age and educational level at childbirth, maternal and paternal type 1 diabetes diagnosed before childbirth

^b^At childbirth

^c^Diagnosed before childbirth

**Table 3:** Evaluation of potential effect-modification by mode of delivery on the association between redemption of antibiotics within the first two years of life and subsequent onset of childhood type 1 diabetes (2 to 14 years, both included).

	Exposed children	Un-exposed children^a^	Partly adjusted	Fully adjusted
	Number of childhood type 1 diabetes		Model^b^	Model^c^
	(person-years at risk)		HR (95% CI)	HR (95%)
**Model evaluating the association with any type of antibiotics in**				
Children born by vaginal delivery				
** **Exposed vs. un-exposed^a^	903 (3,555,860)	357(1,405,814)	0.99 [0.88;1.12]	0.99 [0.87;1.12]
Children born by intrapartum cesarean section				
** **Exposed vs. un-exposed^a^	97 (345,814)	22 (117,784)	1.49 [0.94;2.37]	1.48 [0.92;2.38]
Children born by prelabor cesarean section				
** **Exposed vs. un-exposed^a^	107 (364,335)	17 (116,462)	2.00 [1.20;3.33]	1.91 [1.14;3.20]
**Model evaluating the association with narrow-spectrum antibiotics in**				
Children born by vaginal delivery				
** **Exposed vs. un-exposed^a^	657 (2,600,035)	603 (2,361,639)	0.98 [0.87;1.09]	0.99 [0.88;1.10]
Children born by intrapartum cesarean section				
** **Exposed vs. un-exposed^a^	69 (254,976)	50 (208,622)	1.11 [0.77;1.60]	1.19 [0.81;1.72]
Children born by prelabor cesarean section				
** **Exposed vs. un-exposed^a^	77 (268,268)	47 (212,529)	1.27 [0.89;1.83]	1.25 [0.87;1.80]
**Model evaluating the association with broad-spectrum antibiotics in**				
Children born by vaginal delivery				
** **Exposed vs. un-exposed^a^	651 (2,494,360)	609 (2,467,315)	1.06 [0.95;1.18]	1.05 [0.94;1.18]
Children born by intrapartum cesarean section				
** **Exposed vs. un-exposed^a^	80 (249,034)	39 (214,564)	1.77 [1.21;2.60]	1.70 [1.15;2.51]
Children born by prelabor cesarean section				
** **Exposed vs. un-exposed^a^	82 (266,914)	42 (213,883)	1.57 [1.08;2.28]	1.63 [1.11;2.39]

Hazard ratio (HR) for type 1 diabetes diagnosed in children before 15 years of age are reported with 95% confidence interval (95% CI). Estimates are calculated from the interaction-term between mode of delivery and redemption of antibiotics within the first two years of life in three models evaluating the effect of: redemption of any kind of antibiotics, redemption of narrow-spectrum antibiotics and redemption of broad-spectrum antibiotics, respectively, during the first two years of life.

^a^Defined as: no antibiotics, no narrow-spectrum antibiotics, no broad-spectrum antibiotics, respectively

^b^Partly adjusted model adjusted for: Year of birth, mode of delivery,redemption of antibiotics within the first two years of life, an interaction-term between mode of delivery and redemption of antibiotics within the first two years of life.

^c^Fully adjusted model adjusted for variables above and: sex, parity, maternal and paternal age and educational level at childbirth, maternal and paternal type 1 diabetes diagnosed before childbirth

Based on the absolute risk difference attributed to broad-spectrum antibiotics the number needed to harm were 2,218 in all children regardless of delivery mode, 433 in children delivered by intrapartum cesarean section and 562 in children delivered by prelabor cesarean section.

The association between broad-spectrum antibiotics and childhood type 1 diabetes was not modified by genetic predisposition (maternal or paternal type 1 diabetes diagnosed before childbirth) (P-value for the interaction term between broad-spectrum antibiotics and genetic predisposition: 0.66), parity (P-value for the interaction term: 0.70), maternal redemption of antibiotics during pregnancy (P-value for the interaction term: 0.76) or maternal redemption of antibiotics during the first six months after delivery (P-value for the interaction term 0.78).

We found no indication that the risk of type 1 diabetes was increasing with increasing numbers of antibiotic prescriptions (S4 Table).

When entering redemption of broad-spectrum antibiotics during pregnancy to the fully adjusted model it did not change estimates for the association between broad-spectrum antibiotic treatment of the child and subsequent development of childhood type 1 diabetes; and redemption of broad-spectrum antibiotics during pregnancy was not a predictor of childhood type 1 diabetes (S5 Table).

In analyses including exposure from antibiotic sub-categories, 50.9% of the children had been treated with extended-spectrum penicillin before the age of 2 years and these children had an increased rate of childhood type 1 diabetes (HR 1.12; 95% CI 1.01 to 1.25). Use of macrolides (used by 13.2% of the children) was also associated with an increased rate of childhood type 1 diabetes (HR 1.16; 95% CI 1.01 to 1.34), whereas treatment with combined penicillins, including beta-lactam inhibitors (used by 2.9% of the children) and beta-lactamase sensitive penicillins (used by 44.6% of the children) was not (HR 0.89; 95% CI 0.61 to 1.29 and HR 0.99; 95% CI 0.99 to 1.10, respectively).

## Discussion

Redemption of broad-spectrum antibiotics during the first two years of life was associated with an increased risk of childhood type 1 diabetes in children delivered by cesarean section. In analyses of specific categories of antibiotics, increased risk of childhood type 1 diabetes was found in offspring treated with extended-spectrum penicillins and macrolides.

### Other studies

Associations between use of antibiotics and subsequent development of childhood type 1 diabetes have been evaluated in three other human observational studies. A case-control study from Finland including 437 children diagnosed with type 1 diabetes and 1,748 matched controls, found that overall, there was no association between antibiotic use before pregnancy, during pregnancy or during childhood and the risk of childhood type 1 diabetes. However, when evaluating effects of subgroups of antibiotics as well as several potential sub-comparisons combining antibiotic exposure before, during and after pregnancy, they found an increased risk of childhood type 1 diabetes in children from mother-child pairs, where macrolides were used both by the mother before pregnancy and by the child compared to pairs where neither used macrolides (OR 1.76, 95% CI 1.05–2.94). Furthermore, children born to mothers treated with phenoxymethyl penicilins (OR 1.70, 95% CI 1.08 to 2.68) or quinolone antimicrobials (OR 2.43, 95% CI 1.16 to 5.10) one year prior to pregnancy had increased risk of type 1 diabetes. The use of more than seven purchases of antibiotics during childhood was associated with increased risk of type 1 diabetes in the child (OR 1.66, 95% CI 1.24 to 2.24), but no dose-response effect was observed in the antimicrobial sub-groups. The study did not account or correct for the number of included comparisons. [11] A Dutch case-control study including 925 individuals with type 1 diabetes and 3,591 controls found that patients in the type 1 diabetes group received more antibacterials than controls (49.8 vs. 40.0%, P<0.001), and that the number of anti-infective prescriptions were higher in the type 1 diabetes group from eight years before until four years after the diagnosis.[10] A previous Danish cohort study including all children born in Denmark 1995–2003, hereof 454 children diagnosed with type 1 diabetes, found no statistically significant association between overall use of antibiotics during childhood and risk of type 1 diabetes (rate ratio 1.16, 95% CI 0,91 to 1,50). No specific class of antibiotics (extended-spectrum penicillin, beta-lactamase sensitive penicillins, macrolides or other systemic antibiotics) was associated with type 1 diabetes, no specific age of use was associated with type 1 diabetes and no specific age of onset was associated with antibiotic use. Hospitalization with serious bacterial infections was not associated with increased risk of type 1 diabetes. The study did not explore potential effects of mode of delivery.[9]

### Strengths and limitations

The Danish registers provide unique data as they are nationwide, almost complete and with high validity.[16–20] The prospectively collected data limits the risk of selection and information bias. It is a strength of the study that antibiotics are only sold on prescription and redemptions are automatically transferred to the Register of Medicinal Product Statistics.[19]

No other studies have explored the potentially modifying effect of delivery mode, parity and genetic predisposition or focused on the increasing use of broad-spectrum antibiotics and the present study is the largest with the longest follow-up time. Despite the rising incidence childhood type 1 diabetes is still a rare disease, but it is associated with an increased morbidity.[24] Studies have shown that up to 50% of all antibiotic prescriptions could have been omitted,[25] and that the cesarean section rates are much higher than medically indicated in many countries.[26] Therefore a number needed to harm of around 500 in children delivered by cesarean section is of clinical importance.

Antibiotics can be categorized as either narrow- or broad-spectrum antibiotics according to the spectrum of activity, but there is no universal algorithm for this categorization,[27] and the categorization is complicated as the spectrum of activity changes due to changes in the resistance patterns over time. Traditionally, bacteria are categorized using the Gram stain. Antibiotics only active either against Gram-positive or Gram-negative bacteria have a narrow spectrum whilst antibiotics active against both groups have a broad spectrum.[21;28] We chose the Danish antibiotics categorization which is well defined and founded on considerations of the national resistance patterns,[21] but other categorizations have been suggested.[29–31] We found an increased risk of childhood type 1 diabetes in children exposed to extended-spectrum penicillins and macrolides, of which the first is considered broad-spectrum following the DANMAP categorization but narrow-spectrum following other categorizations and the latter the opposite way around. This finding underlines the potential association between antibiotic treatment during childhood and later childhood type 1 diabetes, but it also stresses the challenges associated with the categorization into broad-and narrow-spectrum antibiotics.[21;29–31] We focused the analyses on the potential association between treatment with antibiotics within the first two years of life, after which the microbiota has been found to be indistinguishable from the adult microbiota.[2] We furthermore focused on broad-spectrum antibiotics, well-knowing that 31% of the children had redeemed both narrow- and broad-spectrum antibiotics. No dose-response effects were found.

Unfortunately, we did not have access to information on the underlying infection or antibiotic treatment during hospitalization. However, missing information regarding patient compliance and antibiotic treatment during hospitalization dilute the effect and underestimate the true effect of antibiotics, and a previous Danish study including information regarding hospitalization with serious bacterial infection did not find an effect of this exposure on type 1 diabetes risk.[9] It is a further limitation, that we did not have access to information regarding ethnic origin, infant diet or breastfeeding.

The predictive validity of type 1 diabetes diagnoses based on discharge codes from the National Patient Registry or on redeemed insulin prescriptions was in 1996 estimated to be 96% in the general population and in 2007 to be 94% to 97% in children.[32;33] There are several potential pathogenic explanations to the findings of an association between redemption of broad-spectrum antibiotics within the first two years of life and increased risk of childhood type 1 diabetes only in children delivered by cesarean section.

Firstly, it may be a causal association with broad-spectrum antibiotics. The pathogenesis of type 1 diabetes is supposed to be multifactorial, with a strong genetic predisposition influenced by several intrauterine, perinatal and postnatal triggers. Children delivery by cesarean section has a less diverse and more pathogenic microbiota,[34] which combined with a prolonged recovery phase following treatment with broad-spectrum antibiotics may result in eradication of harmless and beneficial types of bacteria and overgrowth of more pathogenic bacteria, introducing an imbalance in the microbiota and disturbance of the immunological maturation. This “hygiene theory” has been proposed to be a potential etiology for increased autoimmune activity.[1;2]

Secondly, it has been proposed that delivery by cesarean section may in itself weaken the baby´s immune system through a reduced stress-response. Combined with a more pathogenic gut-microbiota caused by broad-spectrum antibiotics this may induce autoimmune activity or accelerate autoimmune processes.[2;35]

Thirdly, it may not be a causal association with broad-spectrum antibiotics but rather an association with the underlying infection which may trigger autoimmunity. Studies show inconsistent results.[1;36;37] The findings of the association only among children delivered by cesarean section indicate a “two-hit” pathogenesis, that these children are vulnerable to harmful effects of either infections or side-effects from broad-spectrum antibiotics.

Finally, the association may reflect that patients with diabetes have more bacterial infections and therefore redemption of broad-spectrum antibiotics before onset of diabetes could act as a proxy for sub-clinical diabetes.[38] However, then it would be expected that similar associations were found irrespective of mode of delivery.

We found a comparable association with broad-spectrum antibiotics in children delivered by prelabor and intrapartum section. This could indicate that the coding of cesarean section is invalid, that the microbiota is equally affected from intrapartum and prelabor cesarean section or that the interaction with cesarean section is related to shared factors like anesthesia, operative procedure, a reduced fetal stress response or antibiotics given in relation to the operation. Additionally, intrapartum antibiotics would have been given to a proportion of women having intrapartum cesarean section, which could in theory disturb the gut microbiota of these children more than the disturbance attributed to cesarean section per se. Unfortunately, we did not have access to information regarding intrapartum antibiotic treatment.

The finding that paternal and maternal type 1 diabetes are strong predictors of type 1 diabetes is known from other studies. We found, that children of mothers with a short education had a higher risk of type 1 diabetes. Other studies have found diverging associations between maternal educational level and the risk of type 1 diabetes in the children. The finding of a higher risk of type 1 diabetes in firstborn children may be supportive of the “hygiene hypothesis” as they would not have been exposed to microbes from siblings.

## Conclusions

Redemption of broad-spectrum antibiotics during the first two years of life is associated with an increased risk of childhood type 1 diabetes, however, strongly modified by mode of delivery, as the association was only observed in children delivered by cesarean section. The finding may reflect a causal association with either broad-spectrum antibiotics or the underlying condition leading to the treatment and that the association is modified by conditions associated with delivery by cesarean section. Treatment with extended-spectrum penicillins and macrolides may confer specific risk.

## Supporting Information

S1 Table Specification of registers and codes used for defined variables. (DOCX)

S2 Table Classification of types of antibiotics into narrow- or broad-spectrum in accordance with the DANMAP definition. (DOCX)

S3 Table Associations between redemption of broad-spectrum antibiotics within the first two years of life and subsequent onset of childhood type 1 diabetes (2 to 14 years, both included). Effects of birth weight and gestational age.
(DOCX)

S4 Table Associations between redemption of broad-spectrum antibiotics within the first two years of life and subsequent onset of childhood type 1 diabetes (2 to 14 years, both included). Dose-response effects.
(DOCX)

S5 Table Associations between redemption of broad-spectrum antibiotics within the first two years of life and subsequent onset of childhood type 1 diabetes (2 to 14 years, both included). Effects of broad-spectrum antibiotics during pregnancy.
(DOCX)
