# Supplementary material for: Broad-Spectrum Antibiotic Treatment and Subsequent Childhood Type 1 Diabetes: A Nationwide Danish Cohort Study
Source: PLoS One. 2016 Aug 25;11(8):e0161654. doi: 10.1371/journal.pone.0161654 (PMC4999141; doi:10.1371/journal.pone.0161654)
Supplement: S1 Table — (DOCX) [file pone.0161654.s001.docx]

| **S1 Table. Specification of registers and codes used for defined variables** | |  |
| --- | --- | --- |
| **Variable** | **Code** | **Register** |
| **Cesarean section** |  |  |
| *Intrapartum cesarean section^a^* | OP: KMCA00, KMCA10D, KMCA10E, KMCA12, KMCA12A, | MBR, NPR |
|  | KMCA12B, KMCA20, KMCA30, KMCA33, KMCA96 |  |
| *Prelabor cesarean section* | OP: KMCA10, KMCA10A, KMCA10B, KMCA10C, KMCA11 | MBR, NPR |
| **Type 1 diabetes** | ICD8: 249 | NPR |
|  | ICD10: DE10, DP702 | NPR |
| **Unspecified diabetes** | ICD8: 250 (before 1987) | NPR |
|  | ICD10: DE12, DE13, DE14, DH360 | NPR |
| **Insulin** | ATC-code: A10A | RMP |
| **Oral antidiabetics** | ATC-code: A10B | RMP |
| Abbreviations: |  |  |
| ATC: Anatomical Therapeutic Chemicals according to WHO | |  |
| ICD8: the International Classification of Diseases, 8^th^ revision, from 1977 to 1993 | |  |
| ICD10: the International Classification of Diseases, 10^th^ revision, since 1994 | |  |
| MBR: Medical Birth Registry since 1997 |  |  |
| NPR: National Patient Registry since 1977 | |  |
| OP: “Nordic Classification of Surgical procedures” since 1996 | |  |
| RMP: The Register of Medicinal Product Statistics since 1997 | |  |
| ^a^Includes cesarean section, where timing regarding onset of labor could not be distinguished | |  |
