# Supplementary material for: Broad-Spectrum Antibiotic Treatment and Subsequent Childhood Type 1 Diabetes: A Nationwide Danish Cohort Study
Source: PLoS One. 2016 Aug 25;11(8):e0161654. doi: 10.1371/journal.pone.0161654 (PMC4999141; doi:10.1371/journal.pone.0161654)
Supplement: S2 Table — (DOCX) [file pone.0161654.s002.docx]

| **S2 Table. Classification of types of antibiotics into narrow- or broad-spectrum** | | | |
| --- | --- | --- | --- |
| **in accordance with the DANMAP definition** | |  |  |
| **ATC-code** | **Generic name** | **DANMAP definition** | |
|  |  | **narrow** | **broad** |
| **A07AA** | **Antibiotics** |  |  |
| A07AA09 | vancomycin (oral) | x |  |
| **J01A** | **Tetracyclines** |  |  |
| J01AA01 | doxycycline |  | x |
| J01AA04 | lymecycline |  | x |
| J01AA07 | tetracyclines |  | x |
| J01AA12 | tigecycline |  | x |
| **J01CA** | **Penicillins with extended spectrum** |  |  |
| J01CA01 | ampicillin |  | x |
| J01CA02 | pivampicillin |  | x |
| J01CA04 | amoxicillin |  | x |
| J01CA08 | pivmecillinam |  | x |
| J01CA11 | mecillinam |  | x |
| **J01CE** | **Beta-lactamase sensitive penicillins** |  |  |
| J01CE01 | benzylpenicillin | x |  |
| J01CE02 | phenoxymethylpenicillin | x |  |
| **J01CF** | **Beta-lactamase resistant penicillins** |  |  |
| J01CF01 | dicloxacillin | x |  |
| J01CF05 | flucloxacillin | x |  |
| **J01CR** | **Combinations of penicillins, incl. beta-lactamase inhibitors** |  |  |
| J01CR02 | amoxicillin-clavulanic acid |  | x |
| J01CR05 | piperacillin-tazobactam |  | x |
| **J01DB** | **First-generation cephalosporins** |  |  |
| J01DB01 | cephalexine | x |  |
| **J01DC** | **Second-generation cephalosporins** |  |  |
| J01DC02 | cefuroxime |  | x |
| **J01DD** | **Third-generation cephalosporins** |  |  |
| J01DD01 | cefotaxime |  | x |
| J01DD02 | ceftazidime |  | x |
| J01DD04 | ceftriaxone |  | x |
| **J01DF** | **Monobactams** |  |  |
| J01DF01 | aztreonam | x |  |
| **J01DH** | **Carbapenems** |  |  |
| J01DH02 | meropenem |  | x |
| J01DH03 | ertapenem |  | x |
| **J01EA** | **Trimethoprim** |  |  |
| J01EA01 | trimethoprim | x |  |
| **J01EB** | **Sulfonamides** |  |  |
| J01J01EB02 | sulfamethizole | x |  |
| **S2 Table** | **(continued)** |  |  |
| **J01EE** | **Sulfonamides + Trimethoprim** |  |  |
| J01EE01 | sulfamethoxazole + trimethoprim |  | x |
| **J01FA** | **Macrolides** |  |  |
| J01FA01 | erythromycin | x |  |
| J01FA06 | roxithromycin | x |  |
| J01FA09 | clarithromycin | x |  |
| J01FA10 | azithromycin | x |  |
| **J01FF** | **Lincosamides** |  |  |
| J01FF01 | clindamycin | x |  |
| **J01G** | **Aminoglycosides** |  |  |
| J01GB01 | tobramycin |  | x |
| J01GB03 | gentamicin |  | x |
| **J01M** | **Fluoroquinolones** |  |  |
| J01MA01 | ofloxacin |  | x |
| J01MA02 | ciprofloxacin |  | x |
| J01MA14 | moxifloxacin |  | x |
| **J01XA** | **Glycopeptides** |  |  |
| J01XA01 | vancomycin (intravenous) | x |  |
| J01XA02 | teicoplanin | x |  |
| **J01XB** | **Polymyxins** |  |  |
| J01XB01 | colistinmethatnatrium |  | x |
| **J01XC** | **Steroid antibacterials** |  |  |
| J01XC01 | fusidic acid | x |  |
| **J01XD** | **Imidazole derivates** |  |  |
| J01XD01 | metronidazole (intravenous) | x |  |
| **J01XE** | **Nitrofuran derivates** |  |  |
| J01XE01 | nitrofurantoin | x |  |
| **J01XX** | **Other antibacterials** |  |  |
| J01XX08 | linezolide | x |  |
| J01XX09 | daptomycin | x |  |
| **J04AB** | **Drug for treatment of tuberculosis** |  |  |
| J04AB02 | rifampicin | x |  |
| J04AB04 | rifabutin | x |  |
| **P01AB** | **Nitroimidazole derivatives** |  |  |
| P01AB01 | metronidazole (oral, rectal) | x |  |
| DANMAP: Danish Integrated Antimicrobial Resistance Monitoring and Research Programme | |  |  |
