# Supplementary material for: Broad-Spectrum Antibiotic Treatment and Subsequent Childhood Type 1 Diabetes: A Nationwide Danish Cohort Study
Source: PLoS One. 2016 Aug 25;11(8):e0161654. doi: 10.1371/journal.pone.0161654 (PMC4999141; doi:10.1371/journal.pone.0161654)
Supplement: S4 Table — Dose-response effects. (DOCX) [file pone.0161654.s004.docx]

| **S4 Table. Associations between redemption of broad-spectrum antibiotics within the first two years of life and subsequent onset of childhood type 1 diabetes (2 to 14 years, both included). Dose-response effects.** | | | |
| --- | --- | --- | --- |
|  |  | **Children who within the first two years of life redeemed** | |
|  |  | **Broad-spectrum** | **Broad-spectrum** |
|  |  | **Antibiotics** | **Antibiotics** |
|  |  | **Fully adjusted model^a^** | **Fully adjusted model^a^** |
| **Variable** | | **HR (95% CI)** | **HR (95% CI)** |
| **Number of children** | | 810,946 | 810,946 |
| **Number of observed events** | | 1,442 | 1,442 |
| **Redemption of broad-spectrum antibiotics** | |  |  |
|  | **No** | 1.00 |  |
|  | **Yes** | 1.13 [1.02;1.25] |  |
| **Number of redemptions of broad-spectrum antibiotics** | |  |  |
|  | **0** |  | 1.00 |
|  | **1** |  | 1.11 [0.97;1.27] |
|  | **2 or more** |  | 1.14 [1.01;1.29] |
| **Mode of delivery** | |  |  |
|  | **Vaginal** | 1.00 | 1.00 |
|  | **Intrapartum cesarean section** | 0.96 [0.79;1.17] | 0.96 [0.79;1.17] |
|  | **Prelabor cesarean section** | 0.99 [0.81;1.19] | 0.99 [0.81;1.19] |
| **Sex** | |  |  |
|  | **Female** | 1.00 | 1.00 |
|  | **Male** | 0.93 [0.84;1.04] | 0.93 [0.84;1.04] |
| **Parity** | |  |  |
|  | **Multiparous** | 1.00 | 1.00 |
|  | **Primiparous** | 1.12 [1.00;1.26] | 1.12 [1.00;1.26] |
| **Paternal age (years)^b^** | |  |  |
|  | **0-24** | 1.05 [0.79;1.40] | 1.05 [0.79;1.40] |
|  | **25-30** | 1.00 | 1.00 |
|  | **31-100** | 0.95 [0.83;1.08] | 0.95 [0.83;1.08] |
| **Maternal age (years)^b^** | |  |  |
|  | **0-24** | 0.99 [0.80;1.22] | 0.99 [0.80;1.22] |
|  | **25-30** | 1.00 | 1.00 |
|  | **31-100** | 1.04 [0.92;1.18] | 1.04 [0.92;1.18] |
| **Paternal education^b^** | |  |  |
|  | **Elementary school/high school** | 1.00 | 1.00 |
|  | **Short education/skilled worker** | 1.02 [0.90;1.16] | 1.02 [0.90;1.16] |
|  | **Medium/long education** | 0.95 [0.80;1.12] | 0.95 [0.80;1.12] |
| **Maternal education^b^** | |  |  |
|  | **Elementary school/high school** | 1.00 | 1.00 |
|  | **Short education/skilled worker** | 1.13 [0.99;1.29] | 1.13 [0.99;1.29] |
|  | **Medium/long education** | 1.02 [0.87;1.20] | 1.02 [0.87;1.20] |
| **Paternal type 1 diabetes^c^** | |  |  |
|  | **No** | 1.00 | 1.00 |
|  | **Yes** | 11.21 [8.85;14.20] | 11.21 [8.85;14.20] |
| **Maternal type 1 diabetes^c^** | |  |  |
|  | **No** | 1.00 | 1.00 |
|  | **Yes** | 6.19 [4.31;8.91] | 6.19 [4.31;8.91] |
| Hazard ratio (HR) for type 1 diabetes diagnosed in children before 15 years of age are reported with 95% confidence | | | |
| interval (95% CI) | |  |  |
| ^a^Fully adjusted model adjusted for: Year of birth, mode of delivery, sex, parity, maternal and paternal age and | | | |
| educational level at childbirth, maternal and paternal type 1 diabetes diagnosed before childbirth | | | |
| ^b^At childbirth | |  |  |
| ^c^Diagnosed before childbirth | |  |  |
